# Effects of media on preventive behaviour during the COVID-19 pandemic

**DOI:** 10.1057/s41599-023-01554-9

**Published:** 2023-02-14

**Authors:** Takahisa Suzuki, Hitoshi Yamamoto, Yuki Ogawa, Ryohei Umetani

**Affiliations:** 1 Tsuda University, Tokyo, Japan; 2 Rissho University, Tokyo, Japan; 3 Ritsumeikan University, Kyoto, Japan; 4 University of Tsukuba, Tsukuba, Japan

**Keywords:** Science, technology and society, Cultural and media studies

## Abstract

The novel coronavirus 2019 (COVID-19) pandemic required implementation of a variety of measures. In addition to pharmaceutical measures, such as vaccines, changing individuals’ nonpharmaceutical preventive behaviour is essential to prevent the spread of infection. In uncertain situations, such as a pandemic, media sources are important for guiding individuals’ decision-making behaviour. In this study, we examined the effects of media use on preventive behaviour during COVID-19. Earlier studies have shown that social networking service (SNS) browsing promotes preventive behaviour. However, those studies only assessed a single point during the early stages of the pandemic; therefore, the effects on ongoing preventive behaviour are unclear. Thus, a two-wave panel survey was conducted in 2020 and 2021 for an exploratory analysis of changes in the effects of media on individuals’ preventive behaviour over time. The results show that the effect of SNS browsing on preventing going out was confirmed only during the early stage of the pandemic and was not observed 1 year later. It is also shown that those who shifted from self-restraint to going out within 1 year were not affected by the type of media use, but by cognitive factors. As the situation changes during a pandemic, analyses that consider time-series changes are essential for gaining insights about the effects of media on the promotion and maintenance of continuous prevention behaviours.

## Introduction

Following the world’s first reported case of the novel coronavirus 2019 (COVID-19) infection, the disease spread globally. During the first 2 pandemic years, the cumulative number of infections was more than 400 million, and there were more than 6 million deaths. Partly because of strain mutations, the necessary response was longer-term than originally anticipated.

Pharmaceutical measures were introduced relatively late to prevent spread of the disease. Prior to that, nonpharmaceutical measures, including wearing masks, handwashing, and social distancing, were the mainstay preventative practices. After their development, vaccines alone cannot completely control the spread of the disease; therefore, nonpharmaceutical measures must also be continued, as needed. Among these measures, social distancing and reducing contact are effective for prevention (Glass et al., 2006; Block et al., 2020; Badr et al., 2020; Koo et al., 2020). Although legally enforceable lockdowns and other measures have been implemented in many areas, their economic costs make strong restraints difficult and limited in the long term. Top-down enforceable measures alone have limitations; therefore, voluntary behaviour change is required to prevent the long-term spread of the virus (Mao, 2011; Yan et al., 2021). Some studies have shown that voluntary preventive behaviour was more important than the effect of enforceable instructions, such as stay-at-home orders (Quaas et al., 2021). Thus, voluntary preventive behaviour will continue to be important.

Studies have also shown that there are individual differences in the extent to which people engage in infection prevention behaviours during a pandemic. For example, compliance depends on some personal traits, such as individual attributes (e.g., age, gender), economic status and political attitudes (Aschwanden et al., 2021; Badr et al., 2020; Barber and Kim, 2021; Clark et al., 2020; Gollwitzer et al., 2020; Weill et al., 2020; Dinic and Bodroza, 2021; Heffner et al., 2021; Howard, 2022; Ingram et al., 2021). Another crucial factor is obtaining media information. The importance of media use in uncertain situations has been shown consistently, not only during pandemics, but also during terrorism events and natural disasters (e.g., Procopio and Procopio, 2007; Palen et al., 2009). Especially as interpersonal contact is limited during a pandemic, media information becomes an even more important source (Anwar et al., 2020). Because of the particular uncertainty in the early days of the COVID-19 pandemic, the degree of preventive action strongly depended on informational contact by individuals and subjective judgements. Fenichel et al. (2013) found that the contents of media communication and individuals’ subjective judgements become more important than objective indicators. They showed that objective risk indicators during a pandemic (i.e., the number of infected people) do not correlate with people’s behaviour in terms of refraining from going out (i.e., the number of airline ticket cancellations). Even during the recent pandemic, media effects should have been significant in decisions to take infection preventive actions, thus a detailed exploration of media effects in this context is needed.

When considering media effects on preventive actions during COVID-19, it is important to note that infection spread has been prolonged and that the situation may have changed significantly over time. Therefore, media effects on preventive actions may also have changed. To investigate media effects, it is necessary to avoid examining the situation at only a single time point. Instead, accumulating findings from a long-term perspective is required to clarify the relationship between media use and infection prevention behaviour during the COVID-19 pandemic.

It is likely that vigilance against the risk of infection will need to continue in the future; therefore, clarification of how media information encourages voluntary preventive behaviour will be important for preventing the ongoing spread of infectious diseases. This study explored media effects on prevention behaviour (i.e., refraining from going out) using data from a panel survey conducted in Japan in 2020 and 2021.

## Media effects and preventive behaviour

In media effects theory (Bennett and Iyengar, 2008; Messing and Westwood, 2014), traditional mass media such as TV and newspapers, and social media such as social networking service (SNS) have different effects on audiences. Therefore, distinguishing media types should be considered when considering differences in individuals’ tendency to engage in preventive behaviour. Mass media is characterised by high reliability and uniform information broadcasting, whereas social media is characterised by low reliability because individuals can easily publish information and are selectively exposed to preferred content by algorithms. Therefore, it is appropriate to pay particular attention to the impacts of social media to examine individual differences in preventive behaviour.

Social media is characterised by facilitating selective exposure to users’ preferred information and in recent years has been evaluated for several issues, such as echo chambers and fake news (Boutyline and Willer, 2017; Törnberg, 2018). An echo chamber is an environment that seems to repeat one’s own opinions and is formed by selectively connecting on social media with only those who hold opinions similar to oneself. Such an environment is also prone to confirmation bias and opinion polarisation that reinforces one’s own predispositions. As a result, within echo chamber environments, people are more likely to misperceive public opinion, making them more likely to believe and more easily spread fake news or misinformation (Del Vicario et al., 2016). Fake news and misinformation disseminated on social media includes conspiracy-related posts that are not based on scientific evidence or posts that are biased towards a particular opinion. Quattrociocchi et al. (2016) analysed both science-based and conspiratorial posts on Facebook, showing that Facebook users tend to promote their favoured narratives and hence form polarised groups. Their findings also showed differences between the two types of information diffusion over time: conspiratorial posts were spread over a long period, while science-based posts were spread instantaneously but then only discussed among specialised groups of experts.

When people feel anxious, they tend to believe and spread information, even that about which they are less certain (Rosnow, 1991); thus, a great deal of diverse information can be circulated on social media in times of emergency. During the COVID-19 pandemic, uncertainty and anxiety about first encountering an unknown disease accelerated the distribution of a variety of information (Cantwell and Kushlev, 2021; Freiling et al., 2021). In this context, not only fake news and misinformation but information about policies and personal opinions about them could influence users’ attitudes towards preventive behaviour. Compared with the USA, in Japan fake news tends to be spread less on social media because traditional mass media such as newspapers and television are still more trusted (Owen et al., 2020). Yet it remains possible that social media use may have affected preventive behaviour there too.

Earlier studies examined the effects of social media on prevention behaviour and pointed out that SNS provide much information about the severity of infectious diseases, which evokes viewers’ negative emotions (Gao et al., 2020; Buchanan et al., 2021; Heffner et al., 2021; Rettie and Daniels, 2021). In addition, this makes SNS viewers feel fearful of going out and they consequently refrain from doing so (Yoo et al., 2016; Liu, 2021; Mahmood et al., 2021; Zeballos Rivas et al., 2021). For example, Mahmood et al. (2021) used the extended parallel process model (EPPM) to show that SNS browsing promotes individuals’ decisions to refrain from going out. According to the EPPM, SNS browsing increases threat perception and self-efficacy, both of which promote refraining from going out. In contrast, other studies have shown that both social and mass media usage are positively correlated with voluntary preventive intentions (Lin et al., 2014, 2020; Giri and Maurya, 2021). It is still possible that social media use may reduce preventive behaviour because people who are willing to go out but are hesitant to do so may reinforce their intentions by selectively browsing information that encourages them.

Studies analysing SNS contents have shown that there are time periods when posts indicating threats decrease and posts indicating a sense of ease increase (Toriumi et al., 2020). In addition, some contents decrease risk perceptions (Hopfer et al., 2021). It is possible that those who are negative about self-restraint may be selectively exposed to such SNS information and are thus encouraged to go out. Conspiracy theories are also prevalent on social media; those who believe in them tend to rebel against requests for restraint (Enders et al., 2021; Melki et al., 2021; Romer and Jamieson, 2021). These individuals may further strengthen their intentions to resist preventive action by looking for further contents on social media to confirm their bias. Alternatively, it has also been noted that optimism bias is negative for prevention behaviour (Druică et al., 2020). Social media use may reinforce this in the same way. As described above, SNS usage may have the effects of both discouraging and encouraging individuals’ decisions to go out. Therefore, careful analyses are required.

One limitation of the previous studies is that their results were all based on data from a single time point and did not account for changes over time. A major characteristic of the current COVID-19 pandemic is that global infection has continued over the long term. Behaviours, public opinions and perceptions have changed dramatically in this period. During the COVID-19 pandemic, media information has changed over time as social conditions change and media effects on users may also change (Durazzi et al., 2021; Zheng et al., 2021). While media information may encourage preventive behaviour when it is dominated by pessimistic news, it may be less effective or even discourage preventive behaviour at other times. However, most previous studies were based on cross-sectional surveys with a single-dependent variable and thus fail to account for longitudinal variation. The purpose of this study was to shed light on media effects on preventive behaviours. By using panel data, this study also includes an examination of longitudinal changes in preventive behaviour.

## Methods

### Panel survey

This study used panel survey data from two waves in 2020 and 2021 in Japan. While legally enforceable lockdowns were implemented in mainly Western countries, citizens in Japan were requested to voluntarily refrain from leaving their homes without legal restraint. Therefore, Japanese citizens had to decide on their own behaviour based on media information and the behaviours of those around them. In other words, the Japanese data are well suited for analysing the effects of media information on people’s behaviour. In addition to refraining from going out, the Japanese population was requested to wear masks and be vaccinated. Mask-wearing rates have always been high in Japan and vaccination rates rose quickly after they became available. However, voluntarily refraining from going out fluctuated depending on the time of year. Therefore, it is appropriate to focus only on refraining from going out as an indicator of preventive behaviour, which is the dependent variable herein.

Online panel surveys were conducted in April 2020 and April 2021. The infection status, people’s knowledge of the virus and the economic situation changed between the early stages of the COVID-19 pandemic and a year later; therefore, people’s attitudes and media information also changed significantly. The first wave of the survey received valid responses from 2000 people. For the second wave, valid responses were obtained from 987 of the original 2000 respondents (49% response rate). Both surveys were conducted at times when the number of infected persons was increasing and just before a state of emergency was declared. Therefore, in both cases, the decision about whether to refrain from going out was left up to individuals and media information was highly important.

### Dependent variables

First, to categorise the respondents in terms of their attitudes toward refraining from going out, cluster analyses were performed in 2020 and 2021, respectively, using indicators related to coronavirus disease. The cluster analyses were based on 35 variables measured at both waves (Table 1). As judging attitudes toward refraining from going out based only on actual behaviour or individuals’ intentions may result in significant error variance, we used a wide range of variables related to infection. For example, some people had no choice but to go out even though they intended to refrain from doing so, while others did not go out because they had no plans even though they did not intend to refrain from doing so. Classification by only these variables would make it difficult to accurately estimate attitudes. Therefore, by classifying respondents into levels based on multiple, comprehensive variables, it was possible to avoid undue influence by short-term behaviours or intentions. Thus, the classification of attitudes toward refraining from going out was the dependent variable herein.

**Table 1:** Cluster analysis variables.

Stay-home intentions and preventive norms (six items).
Went out for each purpose during previous weekend (six items).
Should refrain from going out or attending outdoor cherry blossom viewing parties based on own opinion, opinion on Twitter or public opinion (three items).
Could people who go out for unnecessary and non-urgent errands be blamed or not (five items).
Predicting how often others will stay at home and risk perception (four items).
COVID-19 knowledge (five items).
Your own opinion on stay-home orders is influenced by mass media, social media or opinions of friends and family (three items).
Opinions of general citizens on stay-home orders are influenced by mass media, social media or opinion of friends and family (three items).

*Description:* All items were asked in the same way in both 2020 and 2021 panel survey waves. Only the knowledge items were modified to match the current situation.

### Independent variables

Eight variables related to media were used as the main independent variables to predict attitudes toward refraining from going out. The question regarding SNS use was focused on Twitter because of the large number of Twitter users in Japan. In addition to the frequency of media use, questions included indicators related to media use, such as political interest (indicating the degree of hard news usage), the degree of selective exposure, media suspicion and subjective media literacy. The details of each variable are in Table 2.

**Table 2:** Independent variables.

Variables	Description
Frequency of mass media use	Simple addition of frequencies of using four types of media (e.g., newspapers, TV, news sites operated by newspapers and TV stations, Yahoo! News).
Frequency of social media use	Simple addition of frequencies of using six types of social media (e.g., Facebook, Twitter, LINE, YouTube, other news sites, summary sites/blog).
Media literacy	Simple addition of five measures about the subjective belief of being able to correctly judge the accuracy of information on mass media.
Media suspicion	Simple addition of three measures about suspecting the validity of information in mass media.
Political interest	The attention respondents gave to political matters.
Selective exposure	The degree to which respondents want to know only favourable information about their interests.
Tweet browsing	Whether respondents usually browse tweets.
Tweet posting	Whether respondents usually post tweets.

## Results

### Cluster analysis

**Fig. 1:**
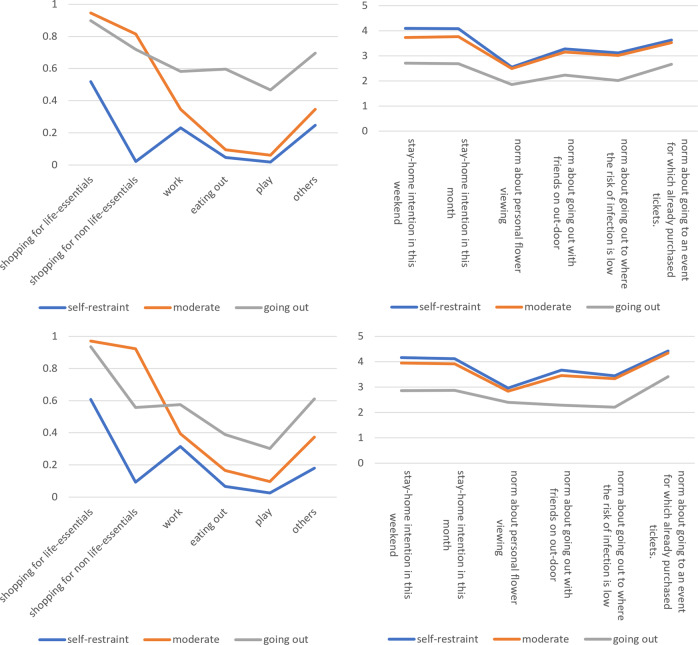
Cluster centres. Larger values are more self-restrained. Top: 2020; Bottom: 2021; Left: Percentage of going out for each purpose during previous weekend; Right: Stay-home intentions and preventive norms.

Using the Ward method, a hierarchical cluster analysis of attitudes toward refraining from going out resulted in three clusters for each year. The centres of the clusters are shown in Fig. 1. In both years, Cluster 1 is classified as the extreme self-restraint group that does not go out for purposes other than shopping for daily necessities and has high self-restraint intentions (hereafter referred to as the ‘self-restraint’ group). Cluster 2 consists of moderates who go out for shopping but not for other purposes and have high self-restraint intentions (hereafter referred to as the ‘moderates’ group). Cluster 3 consists of those who also go out for play, eat out and have low self-restraint intentions (hereafter referred to as the ‘going out’ group). This classification is relative to each point in time and that, overall, refraining from going out behaviour tended to be lower in 2021 than in 2020. Table 3 shows the number of people in each cluster. In 2020, more than half the respondents were in the self-restraint group and 17% were in the going out group, while in 2021, the self-restraint group was 38% and the going out group was 29%, showing a relative increase in the proportion of the going out group.

**Table 3:** Number of people in each cluster.

		2021			
		Self-restraint	Moderate	Going out	Total
2020	Self-restraint	264	165	115	544
	Moderate	79	118	74	271
	Going out	29	47	96	172
	Total	372	330	285	987

### Model selection with stepwise method

Stepwise variable selection was conducted to generate a model to predict attitudes toward refraining from going out. First, we analysed media effects on attitudes toward refraining from going out at each time point using dummy variables for the three attitude categories at each time point as dependent variables, respectively. A stepwise logistic model (decreasing and increasing method using Akaike information criterion as an indicator) was used for model selection. The dependent variables were dummy variables for belonging or not belonging to each cluster and candidate independent variables were media-related variables (see Table 2). Both the 2020 and 2021 independent variables were used for the analysis with 2021 clusters as the dependent variable because the tendency to use media may have changed within individuals, whose lifestyles changed during the pandemic, and because it is difficult to predict in advance whether the independent variables before or after the change will be more effective for predicting attitudes in 2021. The stepwise method made it possible to select variables with predictive power from many candidate variables in an exploratory manner.

**Fig. 2:**
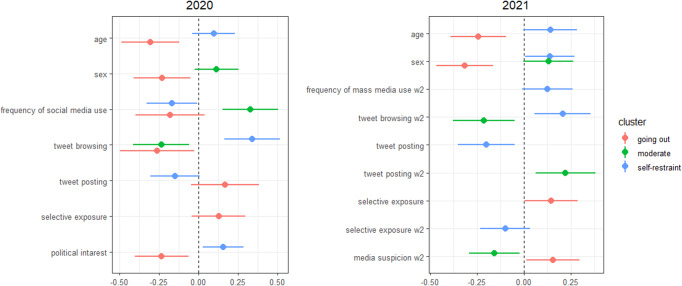
Variables used to predict cluster affiliations at each time point. Left: First wave in 2020; Right: Second wave in 2021. The panels show variables selected by stepwise methods and standardised coefficients with 95% confidence intervals. The variables in the second wave are suffixed ‘w2’, and the variables without suffix are in the first wave.

The variables selected for each year are shown in Fig. 2. With respect to belonging to the self-restraint group, in both 2020 and 2021, tweet browsing had a positive effect and posting had a negative effect. These results replicate the findings from previous studies. On the one hand, this effect is robust in that the same results are obtained over time since the pandemic began. On the other hand, those who belong to the going out group were consistently younger and more likely to be male. In addition to these factors, several aspects of belonging to the going out group were also observed. In 2020, political interest was also important, with high interest tending to lead to self-restraint and low interest tending to lead to going out. In 2021, however, this trend was not seen. This may be because in the early years COVID-19-related news coverage was mostly hard news, whereas after a certain time period the soft news coverage increased. Therefore, even people with low political interest were well informed. In 2021, those with higher levels of media suspicion and selective exposure were more likely to go out. These results show that the features of the self-restraint group did not change dramatically over time, while the features of the going out group did change over time, with the degree of contact with hard news being important in the early stages and the bias of information contact becoming important in the latter stages.

Second, we analysed media effects on the transition between the two time points. As Table 3 shows, about half of all respondents changed their attitudes toward refraining from going out between 2020 and 2021. To predict who maintained the same attitudes and who changed their behaviour from self-restraint to going out, nine dummy variables (3 clusters in 2020 × 3 clusters in 2021) were treated as dependent variables. The candidate independent variables were the same as those used in the 2021 analysis.

**Fig. 3:**
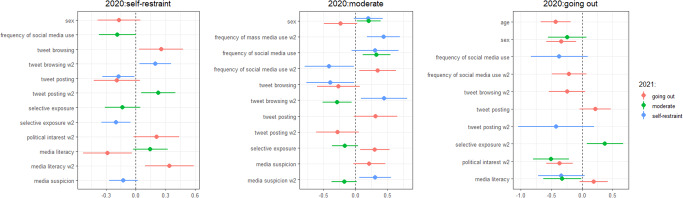
Variables selected for predicting cluster shifts between time points in 2020 and 2021. Left: Self-restraint group; Middle: Moderate group; Right: Going out group. The panels show variables selected by stepwise methods and standardised coefficients with 95% confidence intervals. The variables in the second wave are suffixed ‘w2’, and the variables without suffix are in the first wave.

The variables selected for each transition are shown in Fig. 3. Those who continued to self-restrain in both years browsed tweets, did not post, engaged in less selective exposure and were less suspicious of media. These individuals actively gather information from SNS but in a less biased way. Those who stayed in the going out group in both years were younger, male and less interested in politics. Those who transitioned from self-restraint to going out had high political interest, browsed tweets and had low subjective media literacy in 2020, which increased in 2021. These individuals strived to discern information during the COVID-19 pandemic and may have had actively selective exposure to information that affirmed going out through SNS.

## Discussion

Herein, we examined the effects of media use on attitudes toward refraining from going out over a 2-year period using panel survey data. By using data from two time points, we also analysed time-series changes in the effects of media on attitudes. The results show that use of SNS had a consistent effect on self-restraint attitudes and that in terms of going out attitudes, there were different trends between the initial period and 1 year later. In the early stage of the pandemic, accurate information was difficult to grasp; therefore, proactive efforts to access information, especially hard news, were important for self-restraint attitudes and those who did not actively obtain information went out without understanding the situation. Information had disseminated to some extent after a year; therefore, the difference in proactivity toward information contact disappeared and those with biased information contacts were more likely to go out.

Overall, social media (especially SNS) had stronger predictive power than did mass media use. In addition, the results are remarkably robust against self-restraint. The reason for the absence of an effect of mass media use may be that almost every study participant tended to watch TV because they spent more time at home; thus, a ceiling effect may have made it difficult for individual differences to emerge.

All analyses showed that SNS browsing has a positive effect on self-restraint. However, while SNS browsing had a negative effect on going out during the early period of the COVID-19 pandemic, this effect was not observed 1 year later. One reason for this difference is that those who transitioned from self-restraint to going out tended to view tweets. The results imply that these people may have been using SNS to selectively browse information during the COVID-19 pandemic, thereby fostering going out attitudes.

In contrast to previous studies, which often relied on one-time survey results, this study used panel data, which allowed a more detailed examination. Information content disseminated via media change along with social situations. Therefore, it is inevitable that the effect of media use on attitudes will also change over time. Furthermore, the use of media such as SNS, which enables easy selective exposure to information, may not have a uniform effect. It is necessary to consider differences in the time of year and the effects on self-restraint and going out attitudes, respectively.

This study contributes to the fields of politics, political communication, and sociology. Each of these fields examines policy-relevant information disseminated through the media, and how citizens’ interest in and knowledge about policy is related to policy effectiveness. This study may provide useful insights into the effects of media when government requests depend on voluntary action, as during the COVID-19 pandemic. In addition, attention has been paid to whether social media can cause social conflict and fragmentation. Radical and intolerant SNS posts, by both those who were and those who were not amenable to preventive action, were observed during the COVID-19 pandemic. This study may also provide indications about whether these information sources drive social conflict and fragmentation.

This study was not without some limitations. First, the survey alone cannot reveal concrete media-disseminated information, its time-series changes or the contents to which respondents were exposed. Therefore, content analysis should also be conducted. While this study used a two-wave panel survey, further analyses of long-term changes are also needed.

## Data Availability

The all data of this study are stored in an OSF data package titled Data of ‘Effects of Media on Preventive Behaviour During the COVID-19 Pandemic’, which can be accessed at the below link. 10.17605/OSF.IO/YRBCG.
